# Crossover replantation after bilateral traumatic lower limb amputations: a case report

**DOI:** 10.1186/1752-1947-6-218

**Published:** 2012-07-24

**Authors:** Jun Fang, Huazhuang Li, Honglei Dou, Jingchun Chen, Aiping Xu, Wenguo Liu, Gang Ding

**Affiliations:** 1 First Section, Department of Orthopedics, Yidu Central Hospital, Weifang Medical University, Shandong Province, 262500, PR China; 2 Department of Oral and Maxillofacial Surgery, Yidu Central Hospital, Weifang Medical University, Shandong Province, 262500, PR China

## Abstract

**Introduction:**

Replantation of a limb to the contralateral stump after bilateral traumatic amputations is rare. To the best of our knowledge, there are only a few reports of crossover lower limb replantation in the literature.

**Case presentation:**

We treated a 37-year-old Chinese woman with bilateral lower limb crush injuries sustained in a traffic accident. Her lower limb injuries were at different anatomic levels. We performed emergency bilateral amputations followed by crossover replantation. Five years later, the woman had recovered well, and had perfect movement and stability in her replanted leg. After reviewing the literature, we thought that presentation of our patient’s case might provide useful information for clinicians.

**Conclusions:**

Crossover replantation should be considered when evaluating a patient with bilateral lower limb injuries, thus allowing the patient to touch the ground and stand using their own foot.

## Introduction

Microsurgical techniques have enabled the replantation of traumatically amputated limbs. Crossover replantation or ectopic implantation should be considered in cases of bilateral amputations, to salvage at least one limb [1]. Replantation of a limb to the contralateral stump after bilateral traumatic amputations is rare, and may incur criticism due to the prolonged hospital stay and complications. To the best of our knowledge, there are only a few reports of crossover replantation of the lower limb in the literature [2-5].

## Case presentation

**Figure 1:**
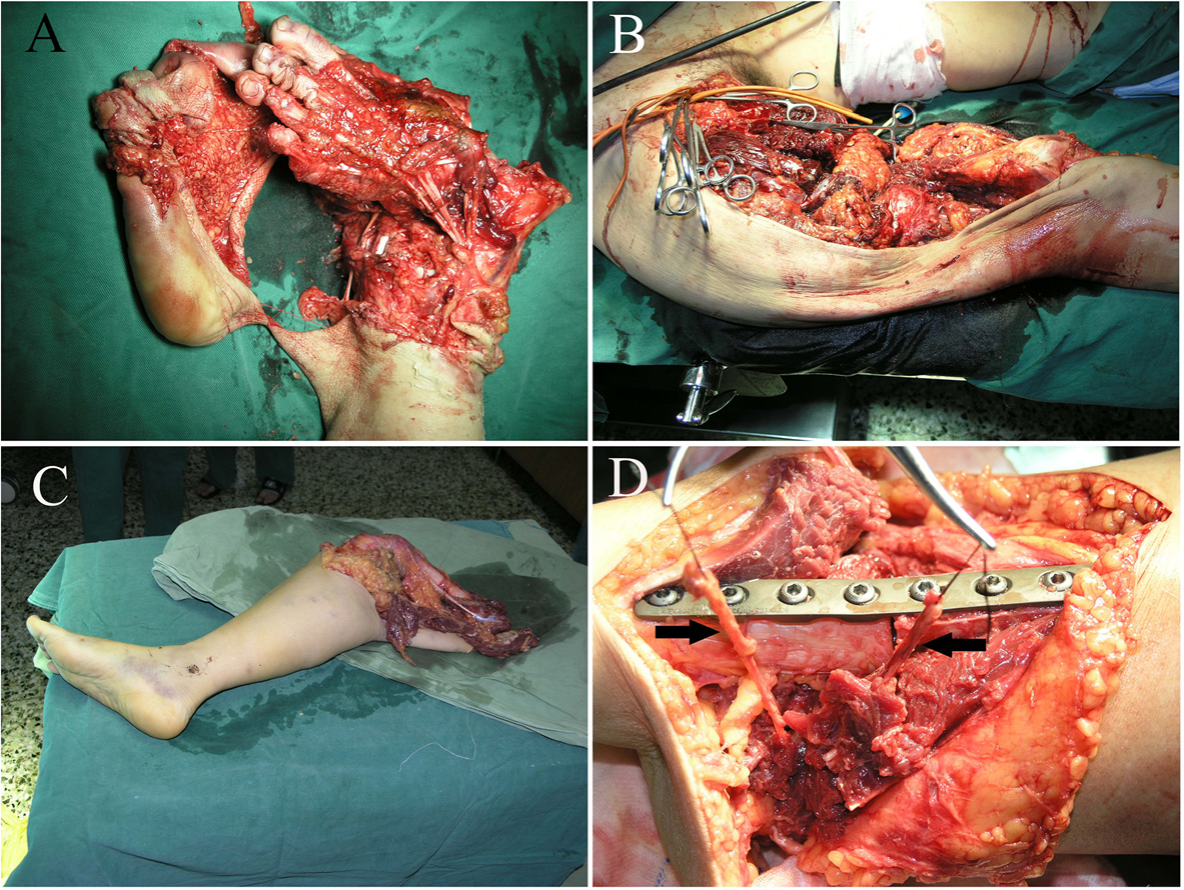
**The status of our patient before and during the operation.** (**A**) The crushed left lower leg. (**B**) The crushed right leg. (**C**) The broken right lower leg. **(D)** Fixation of the right lower leg to the left leg. Left arrow: posterior tibial artery and posterior tibial vein in the right calf. Right arrow: anterior tibial artery and anterior tibial vein in the left calf.

A 37-year-old Chinese woman presented to our department four years and 11 months ago with bilateral lower limb crush injuries sustained in a traffic accident. The lower limb injuries were at different anatomic levels (Figure 1A-C). On the right side, her lower limb was crushed from her hip joint to 16cm below her knee joint, but the bones and soft tissues of the lower one-third of her leg were intact with only slight injury to the skin. On the left side, the distal portion of her leg was crushed. Our patient was in serious hypovolemic shock on arrival, with a heart rate of 150 beats per minute and blood pressure of 80/60mmHg.

After rapid infusion of intravenous fluids, our patient rapidly recovered from shock and did not develop acute renal failure or acute respiratory distress syndrome. Emergency surgery was performed. Bilateral lower limb amputations were necessary. Her lower left leg was unsalvageable, but her lower right leg was suitable for replantation to the left leg stump after debridement. We decided to perform crossover replantation of her right lower leg to the left leg stump to provide our patient with a sensate weight-bearing extremity. Her amputated right lower leg was wrapped in sterile dressings, placed on a sterile tray and stored in the refrigerator at 4°C during fixation of the left leg fracture.

After amputation and debridement of her right hip joint, her right lower tibia was fixed to her left upper tibia (Figure 1D). The fibula was not fixed. The tendons, blood vessels and nerves of her left leg were anastomosed to the amputated lower right leg structures. The anterior tibial artery and posterior tibial artery were anastomosed crosswise, and the ends of the great saphenous vein, small saphenous vein and four deep veins were anastomosed without crossover. The sural nerve and saphenous nerve were anastomosed crosswise, and the anterior and posterior tibial nerves were anastomosed without crossover. Heterotopic replantation of her right lower leg to the left leg stump was thus completed. A stump was created on the right side at her hip joint. Routine antibiotic, anti-coagulant, and anti-angiospasm treatments were administered post-operatively. In a second operation, a soft tissue defect of the replanted limb was covered by a microvascular-free latissimus dorsi muscle flap. The post-operative anti-coagulation regime was as follows: dextran 40 (500mL) twice a day for seven days; aspirin (100mg) orally three times a day for three days; narceine (30mg) four times a day for seven days; and tolazoline (25mg) three times a day for seven days. Routine post-operative blood tests, including coagulation tests, were performed for seven days.

**Figure 2:**
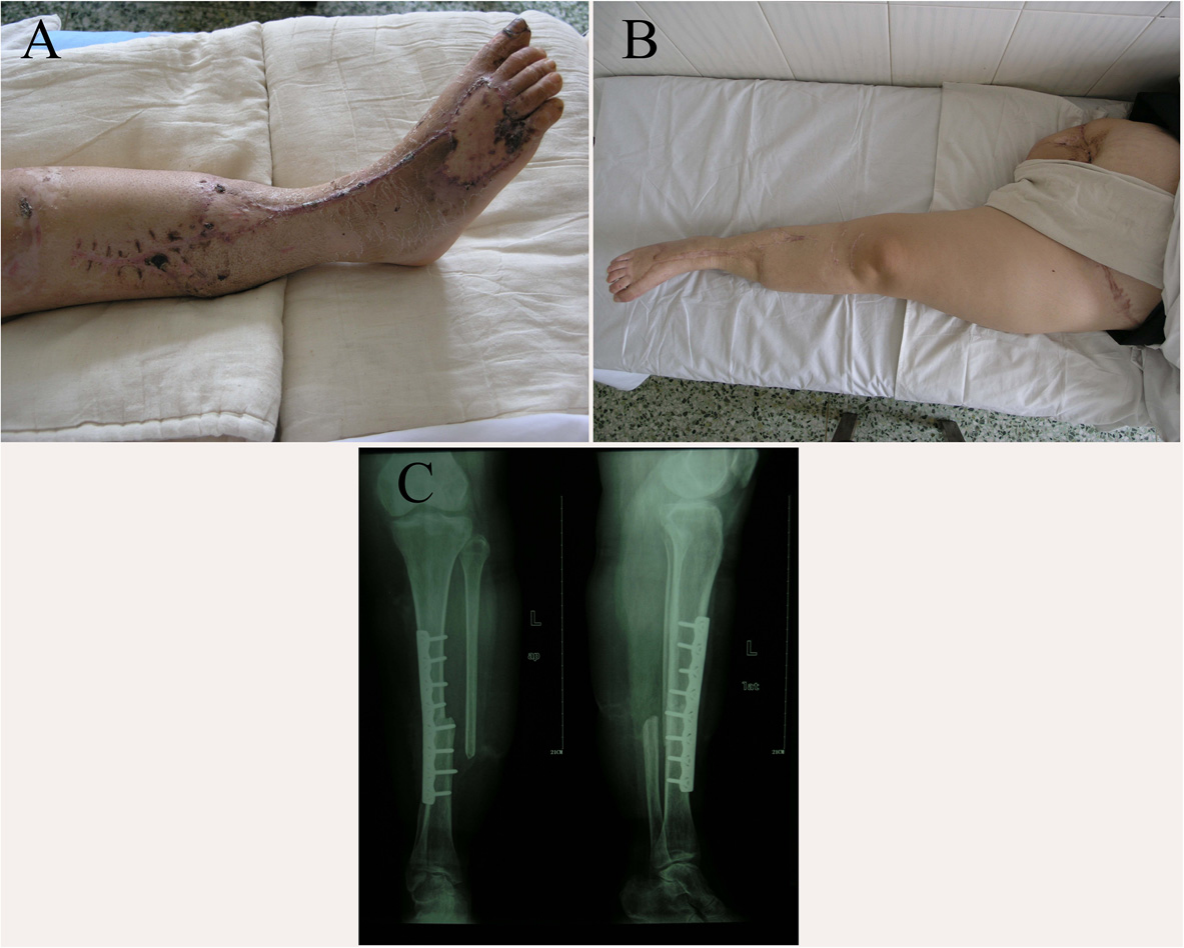
**Post-operative status of our patient.** (**A**) One month post-operatively. (**B**) Three years and three months post-operatively. (**C**) X-ray image taken at 12 months post-operatively.

The replantation was successful and our patient was discharged after two months (Figure 2A). She was rehabilitated with a contralateral prosthesis and ambulates with a walking stick. One year post-operatively, X-ray examination showed perfect union of the tibia (Figure 2C). There was no ulceration of the replanted extremity or the right-sided amputation stump at 39 months post-operatively. The sole of her foot on the left side regained complete protective sensation (Figure 2B). Our patient described the functional result of the replantation as satisfying, and found that the prosthesis on the right side caused more problems than the replanted left lower limb. She had no complaints about the cosmetic result. In addition, she experienced restoration of perceived body height with the crossover replantation.

## Discussion

The indications for replantation depend on many factors, including the general condition of the patient. Although lower leg replantation prolongs hospital stay, delays mobilization and increases the required secondary procedures compared to simple amputation, the functional outcome is much better after replantation than with a prosthetic limb, especially when there is successful restoration of sensation to the weight-bearing area. Function including movement and stability, as well as quality of life, are greatly improved by lower limb replantation.

## Conclusions

The possibility of crossover replantation should be considered when evaluating a patient with bilateral lower limb injuries, thus allowing the patient to touch the ground and stand using their own foot.

## Consent

Written informed consent was obtained from the patient for publication of this case report and any accompanying images. A copy of the written consent is available for review by the Editor-in-Chief of this journal.

## Competing interests

The authors declare that they have no competing interests.

## Authors’ contributions

JF, HL, HD, JC, AX, WL and GD analyzed and interpreted the data from our patient. JF, HL and GD wrote the manuscript. All authors read and approved the final manuscript.
